# Optimizing MatB–MatC-Dependent Malonyl-CoA Supply for Enhanced Raspberry Ketone Production in *Escherichia coli*

**DOI:** 10.3390/biotech15030071

**Published:** 2026-08-21

**Authors:** Kurumi Usui, Naoki Takaya, Shunsuke Masuo

**Affiliations:** 1 Degree Programs in Life and Earth Sciences, Microbiology Research Center for Sustainability, University of Tsukuba, 1-1-1 Tennodai, Tsukuba 305-8572, Japan; s2430256@u.tsukuba.ac.jp; 2 Faculty of Life and Environmental Sciences, Microbiology Research Center for Sustainability, University of Tsukuba, 1-1-1 Tennodai, Tsukuba 305-8572, Japan

**Keywords:** *Escherichia coli*, fed-batch cultivation, malonate, malonyl-CoA, metabolic profiling, raspberry ketone

## Abstract

Raspberry ketone (RK) is a valuable natural flavor compound, but its extraction from plants is inefficient because of its low natural abundance. Microbial production of RK offers a promising alternative; however, insufficient availability of malonyl-CoA can limit RK biosynthesis. In this study, we introduced a malonate-dependent malonyl-CoA supply module into an engineered *Escherichia coli* strain designed for de novo RK production through heterologous expression of malonyl-CoA synthetase MatB and malonate transporter MatC. In the presence of malonate, the introduction of the MatB–MatC module increased RK production by 2.3-fold. To optimize malonate supplementation, RK pathway metabolites, including intracellular acyl-CoA intermediates, were quantified by liquid chromatography–mass spectrometry, and the resulting metabolite profiles were analyzed by principal component analysis, hierarchical clustering, and correlation analysis. This study indicated that 50-mM malonate was optimal, yielding 24 mg/L RK during 96-deep-well plate cultivation. Fed-batch optimization increased RK production to 301 mg/L in a 100-mL jar fermenter, and scale-up cultivation in a 2-L jar fermenter produced 340 mg/L RK. In this study, optimizing MatB–MatC-dependent malonyl-CoA supply, combined with pathway-level metabolic profiling and controlled fed-batch cultivation, enhanced de novo RK production in *E. coli*.

## 1. Introduction

Raspberry ketone (RK) is a natural flavor compound found in plants such as raspberries, grapes, peaches, and rhubarb, and is widely used as a food additive because of its characteristic fruity aroma [1,2]. Although chemically synthesized RK is available at a relatively low cost [3,4,5], naturally derived RK is much more expensive because plants contain only trace amounts of RK, typically 1–4 mg/kg [1,6]. In addition, plant extraction requires large amounts of biomass and raises sustainability concerns [7,8,9]. Therefore, microbial production of RK represents a promising alternative to plant extraction, particularly because fermentation-derived flavor compounds can be regarded as natural flavors in several regulatory contexts [10,11].

Several microbial strategies have been developed for RK production. Early work demonstrated microbial RK biosynthesis by reconstructing plant-derived phenylpropanoid and polyketide pathway reactions in recombinant microorganisms [1]. Subsequent studies have improved RK production through pathway engineering, synthetic enzyme fusion, precursor-based bioconversion, and host metabolic engineering in organisms such as *E. coli*, *Saccharomyces cerevisiae*, and *Corynebacterium glutamicum* [4,12,13,14,15]. In *E. coli*, RK has been produced either by bioconversion from aromatic precursors such as *p*-coumaric acid or by de novo biosynthesis through engineered aromatic amino acid and phenylpropanoid metabolism [12,13,15]. These studies established microbial RK production as a promising platform; however, further improvement in titer, yield, and productivity is still needed. Therefore, precursor supply, pathway balance, and fermentation conditions require further optimization.

**Figure 1:**
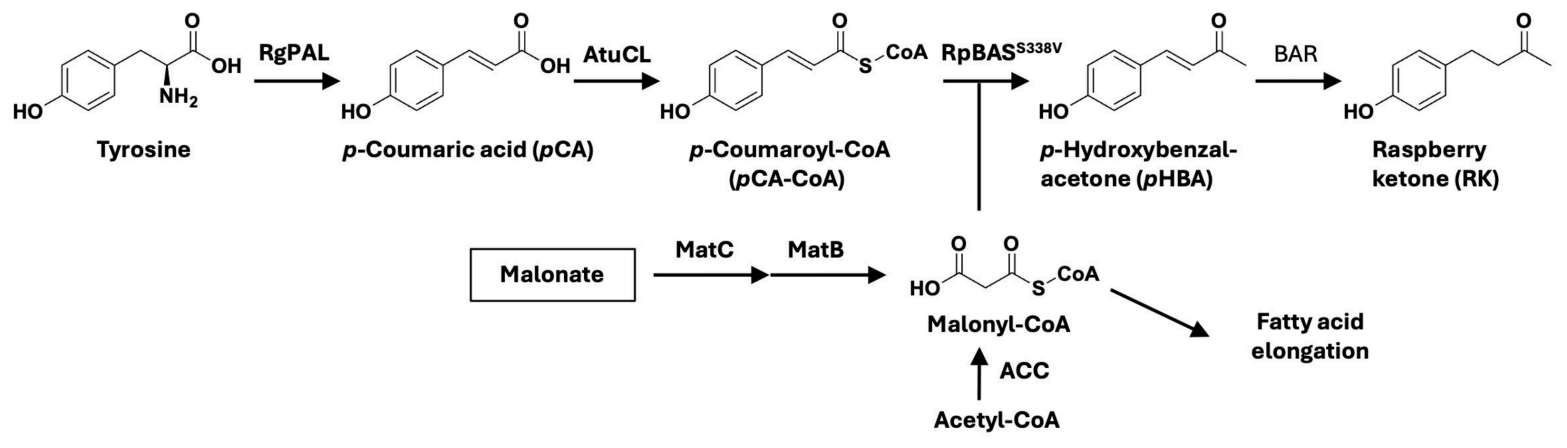
Metabolic pathway for RK biosynthesis and malonyl-CoA supply in engineered *Escherichia coli*. ACC, acetyl-CoA carboxylase; AtuCL, *p*-coumaric acid CoA ligase; BAR, benzalacetone reductase; MatB, malonyl-CoA synthetase; MatC, putative malonate transporter; RgPAL, phenylalanine/tyrosine ammonia-lyase; RK, raspberry ketone; RpBAS^S338V^, benzalacetone synthase.

Benzalacetone synthase (BAS), a plant type III polyketide synthase, catalyzes a key step in the RK biosynthetic pathway [16,17,18]. Specifically, BAS mediates the decarboxylative condensation of *p*-coumaroyl-CoA (*p*CA-CoA) with malonyl-CoA to produce *p*-hydroxybenzalacetone (*p*HBA), which is subsequently reduced to RK by nicotinamide adenine dinucleotide phosphate-dependent benzalacetone reductase (Figure 1) [19,20]. Because BAS requires both *p*CA-CoA and malonyl-CoA as substrates, the availability and balance of these two acyl-CoA precursors strongly influence RK pathway flux. A previous study showed that enhancing *p*CA-CoA production through metabolic engineering of *E. coli* increased RK production, and that supplementation with cerulenin, an inhibitor of fatty acid biosynthesis, further improved RK production by increasing intracellular malonyl-CoA availability [12]. However, the high cost and inhibitory nature of cerulenin limit its practical use. This limitation highlights the need for an alternative strategy to enhance malonyl-CoA supply.

The MatB–MatC system provides a malonate-dependent route for increasing intracellular malonyl-CoA availability. Malonyl-CoA is an essential precursor for fatty acid and polyketide biosynthesis, and its intracellular level is tightly regulated by enzymes involved in malonyl-CoA formation and degradation [21,22,23,24]. In bacterial malonate metabolism, MatB catalyzes the ATP-dependent conversion of malonate to malonyl-CoA, whereas MatC functions as a malonate transporter that mediates malonate uptake [25]. Heterologous expression of *matB* and *matC* has been used to enhance malonyl-CoA supply and improve microbial production of malonyl-CoA-derived compounds, including naringenin and other polyketide-derived products [25,26,27,28]. In addition, *matBC* expression has been reported to increase RK production in *E. coli* [13]. However, in de novo RK-producing strains engineered for aromatic precursor supply, the relationship between MatB–MatC-dependent malonyl-CoA supply, pathway metabolite balance, and final RK production remains insufficiently characterized.

In this study, we introduced a *matBC* expression system into a de novo RK-producing *E. coli* strain previously engineered to increase *p*CA-CoA production but still showing limited final RK yield. We evaluated the effects of *matB* and *matC* expression and malonate supplementation on intracellular malonyl-CoA levels, RK pathway intermediates, and RK production using pathway-level metabolic profiling. We further optimized malonate supplementation and fed-batch cultivation conditions and evaluated RK production in a 2-L jar fermenter. This work provides insight into how MatB–MatC-dependent malonyl-CoA supply affects pathway metabolite balance in a de novo RK-producing *E. coli* strain and supports the utility of this strategy for improving RK production under controlled fermentation conditions.

## 2. Materials and Methods

### 2.1. Strains, Materials, and Instrumentation

Table 1 lists the strains used in this study. *E. coli* JM109 and *E. coli* BL21(DE3) (Novagen, Cambridge, MA, USA) were used for plasmid construction and RK production, respectively. Chemical reagents were obtained from Sigma-Aldrich and Wako Chemicals (St. Louis, MO, USA). Plasmids were constructed using KOD One PCR Master Mix (Toyobo, Osaka, Japan), restriction enzymes (Takara, Shiga, Japan), Ligation high Ver. 2 (Toyobo), and NEBuilder HiFi DNA Assembly Master Mix (New England Biolabs, Ipswich, MA, USA). Metabolites were analyzed by liquid chromatography–electrospray ionization–tandem mass spectrometry (LC–ESI–MS/MS) using an LCMS-8045 system (Shimadzu, Kyoto, Japan).

### 2.2. Plasmid Construction

Table 2 lists the plasmids used in this study. The nucleotide sequences of *matB* (GenBank accession no. KF765783), encoding malonyl-CoA synthetase, and *matC* (KF765784), encoding a malonate transporter, both derived from *Rhizobium leguminosarum* bv. *trifolii*, were codon-optimized for *E. coli*, synthesized, and amplified by polymerase chain reaction (PCR) using the primers listed in Table 3. The amplified *matB* fragment was digested using NcoI and HindIII restriction endonucleases and cloned into pETDuet-1 (Novagen) to generate pET-matB. The amplified *matC* fragment was then cloned into pET-matB digested with NdeI and XhoI restriction endonucleases to obtain pET-matB-matC. The *tyrA* gene from pET-tyrA [12] was amplified by PCR using the primers listed in Table 3 and cloned into pRSF-Rgpal [12] digested with NcoI and HindIII, yielding pRSF-Rgpal-tyrA. The *p*CA-CoA ligase gene from *Agrobacterium tumefaciens* [12] was amplified by PCR using the primers AtuCL_fw2_Nterm and AtuCL_rv2_Nterm. The amplified fragment was digested using NdeI and XhoI and ligated into pET-28a digested using the same restriction enzymes, yielding pET28a-AtuCL_Nterm.

### 2.3. Fermentation

Recombinant *E. coli* BL21(DE3) strains were grown as precultures in 3 mL of Lysogeny Broth (LB) medium supplemented with the appropriate antibiotics required for plasmid maintenance. The antibiotics used in this study were ampicillin (50 µg/mL), chloramphenicol (35 µg/mL), kanamycin (50 µg/mL), and streptomycin (50 µg/mL). The appropriate antibiotic combination was also used during strain maintenance, flask-scale cultivation, 96-deep-well cultivation, and fermenter cultivation. For flask-scale fermentation, 2 mL of each preculture was inoculated into 100 mL of fermentation medium supplemented with or without malonic acid at a final concentration of 50 mM. The fermentation medium contained (per L) 10 g glucose, 10 g tryptone, 5 g yeast extract, 12 g Na_2_HPO_4_, 6 g KH_2_PO_4_, 42 g 3-(N-morpholino)propanesulfonic acid (MOPS), 0.5 g NaCl, 10 g (NH_4_)_2_SO_4_, 0.5 g MgSO_4_·7H_2_O, 15 mg CaCl_2_, 50 mg thiamine-HCl, and 2 mL of trace element solution. Cultures were incubated aerobically at 30 °C with shaking at 120 rpm. When the optical density at 600 nm (OD_600_) reached 0.6, isopropyl β-D-thiogalactoside (IPTG) was added to a final concentration of 0.1 mM, and the cultures were incubated at 28 °C for the indicated period.

For cultivation in 96-deep-well plates, 20 µL of preculture was inoculated into 1 mL of fermentation medium containing 0.1-mM IPTG. The plates were incubated aerobically at 28 °C with shaking at 1500 rpm using an M·BR-032P incubator shaker (TAITEC Corporation, Saitama, Japan).

For fermenter cultivation, 70 mL of fermentation medium was added to a 100-mL Fermenter Bio Jr.8 vessel (Biott, Tokyo, Japan), or 1 L of fermentation medium was added to a 2-L BMZ-P fermenter (Biott). For inoculum preparation, the RKmat strain was grown in 3 mL of LB medium at 30 °C for 12–18 h as a primary preculture. Subsequently, 1 mL of the primary preculture was inoculated into 100 mL of LB medium in a 500-mL baffled flask and cultivated at 30 °C for 12–18 h. The resulting preculture was inoculated into 70 mL or 1 L of fermentation medium to an initial OD_600_ of 1. Unless otherwise specified, the 100-mL and 2-L fermenters were aerated at 0.07 and 1 L/min, respectively. IPTG was added to a final concentration of 0.1 mM when the OD_600_ reached 3. When the glucose concentration in the culture broth fell below 10 g/L, a concentrated 50% glucose solution (*w*/*v*; 500 g/L) was supplied as a feed solution using a peristaltic pump. The pH was monitored using an electrode and maintained at 7.0–7.1 by adding 10% NH_4_OH. Dissolved oxygen (DO) was monitored using an electrode and, unless otherwise specified, maintained at 3.1 ppm by controlling the agitation speed.

### 2.4. Preparation of pCA-CoA

*p*CA-CoA was enzymatically prepared using recombinant *p*CA-CoA ligase. *E. coli* BL21 Star(DE3) harboring pET28a-AtuCL_Nterm was precultured in 3 mL of LB medium at 30 °C for 12–18 h. A 1-mL aliquot of the preculture was inoculated into 100 mL of LB medium in a 500-mL baffled flask and cultivated at 37 °C until the OD_600_ reached 0.2–0.5. Protein expression was induced by adding IPTG to a final concentration of 0.1 mM, followed by cultivation at 20 °C for 24 h. The recombinant enzyme was purified by His-tag affinity chromatography as described previously [29].

For *p*CA-CoA synthesis, the reaction mixture contained 30-mM sodium phosphate buffer (pH 7.1), 5 mM MgCl_2_, 2.5 mM ATP, 0.8 mM CoA, and 0.4 mM trans-*p*CA. The reaction was initiated by adding purified *p*CA-CoA ligase and incubated at 25 °C for 30 min. The reaction was terminated by heating at 95 °C for 10 min. The resulting *p*CA-CoA was purified by reversed-phase high-performance liquid chromatography (HPLC) using a TSKgel ODS-100V column with 10-mM ammonium formate buffer (pH 4.5) and methanol as mobile phases. The HPLC-purified *p*CA-CoA fraction was used as the analytical standard for liquid chromatography (LC)–mass spectrometry (MS) quantification of *p*CA-CoA.

### 2.5. Determination of Metabolite Concentrations

Metabolites were separated using an LC system equipped with a 150 × 2.1-mm ACQUITY UPLC CSH™ C18 column (Waters, Milford, MA, USA). Malonate, tyrosine, *p*CA, *p*HBA, and RK were separated using solvent A, consisting of 0.05% formic acid, and solvent B, consisting of acetonitrile. The gradient program was as follows: initial elution using 100% solvent A for 4 min, followed by an 11-min linear gradient to 50% solvent B, which was then held for 1 min. The column was re-equilibrated for 4 min before the next injection.

Malonyl-CoA and *p*CA-CoA were separated in isocratic mode using 5-mM ammonium formate buffer (pH 7.0) containing 50% acetonitrile as the mobile phase. The flow rate was maintained at 0.4 mL min^−1^, and the column temperature was set to 40 °C throughout the analysis.

Multiple Reaction Monitoring transitions were set as follows: *m/z* 103.0 → 59.15 for malonate in negative ion mode; *m/z* 180.05 → 163.15 for tyrosine in negative ion mode; *m/z* 163.05 → 119.05 for *p*CA in negative ion mode; *m/z* 912.0 → 428.0 for *p*CA-CoA in negative ion mode; *m/z* 165.05 → 127.10 for *p*HBA in positive ion mode; *m/z* 854.05 → 303.00 for malonyl-CoA in positive ion mode; and *m/z* 165.10 → 107.10 for RK in positive ion mode.

The dwell time, Q1 pre-bias, collision energy, and Q3 pre-bias were set to 100 ms, 21 V, 32 eV, and 11 V for malonate; 100 ms, 20 V, 14 eV, and 17 V for tyrosine; 100 ms, 17 V, 14 eV, and 21 V for *p*CA; 100 ms, 20 V, 14 eV, and 21 V for *p*CA-CoA; 100 ms, −12 V, −12 eV, and −15 V for *p*HBA; 100 ms, −34 V, −41 eV, and −30 V for malonyl-CoA; and 100 ms, −14 V, −12 eV, and −11 V for RK, respectively. Intracellular acyl-CoA levels were normalized to dry cell weight (DCW) and expressed as nmol/mg-DCW. DCW was estimated from OD_600_ using a conversion factor of 1 OD_600_ = 0.396 g-DCW L^−1^, as previously reported [30].

### 2.6. Data Analysis

Data analysis was performed using Python version 3.12.13, with SciPy version 1.16.3, Matplotlib version 3.10.0, pandas version 2.2.2, NumPy version 2.0.2, and scikit-learn version 1.6.1. The measured levels of tyrosine, *p*CA, *p*HBA, RK, malonyl-CoA, and *p*CA-CoA were used for principal component analysis (PCA), hierarchical clustering analysis, and Pearson’s correlation analysis. Prior to these analyses, each metabolite variable was standardized by Z-score transformation. PCA was performed using scikit-learn. Standardized malonate values were not included in the calculation of principal components and were used only for visualization. The PCA score plot was generated using the first and second principal components. In the score plot, symbol color represents the standardized malonate value, and symbol size represents the standardized RK value. Hierarchical clustering analysis was performed using Ward’s linkage method with Euclidean distance. Clustering was applied to both the experimental conditions and metabolites, and the results were visualized as a heatmap with dendrograms. Pearson’s correlation coefficients were calculated for all pairwise combinations of metabolites. The results were visualized as a lower-triangular correlation heatmap. Statistical significance was indicated as follows: * *p* < 0.05, ** *p* < 0.01, and *** *p* < 0.001.

## 3. Results

### 3.1. Heterologous Expression of MatB and MatC Increased Intracellular Malonyl-CoA in the Presence of Malonate

**Figure 2:**
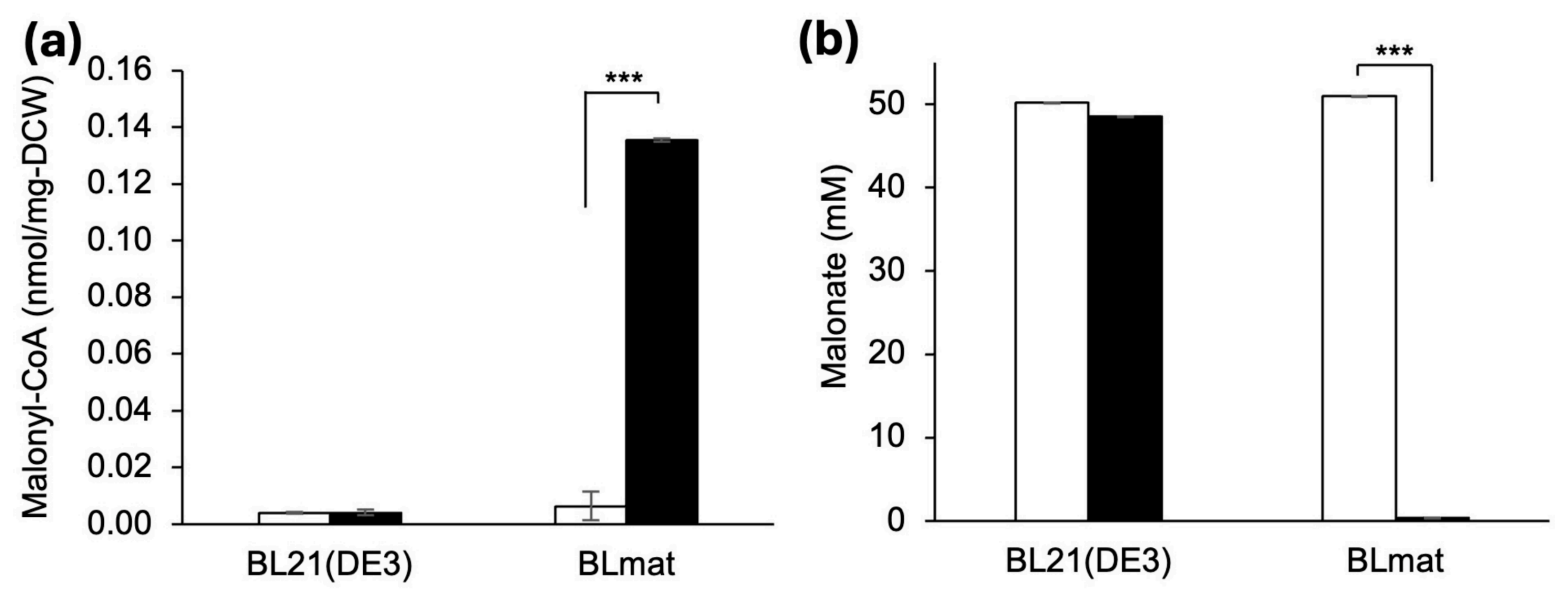
Heterologous expression of *MatB* and *MatC* increased intracellular malonyl-CoA levels and promoted malonate consumption in *E. coli*. *E. coli* BL21(DE3) and the BLmat strain harboring pETDuet-*matB*-*matC* were cultured in LB medium with or without 50-mM malonate for 72 h. (**a**) Intracellular malonyl-CoA levels. White and black bars indicate cultivation without and with 50-mM malonate supplementation, respectively. (**b**) Malonate consumption during cultivation, as determined by measuring the malonate concentration in the culture supernatant at 0 and 72 h. Values represent the mean ± standard deviation of three independent biological replicates. Statistical significance was evaluated using Welch’s *t*-test for the indicated comparisons. Three asterisks indicate *p* < 0.001. BL21(DE3), *E. coli* BL21(DE3); BLmat, BL21(DE3) harboring pETDuet-*matB*-*matC*; DCW, dry cell weight; LB, Lysogeny Broth.

We first constructed *E. coli* strains expressing *matB* and *matC* to examine their effects on malonyl-CoA production in *E. coli*. *E. coli* BL21(DE3) was transformed using pETDuet-matB-matC to generate the BLmat strain. BL21(DE3) and the BLmat strain were cultured in LB medium with or without 50-mM malonate for 72 h in shake flasks. In BL21(DE3), intracellular malonyl-CoA levels remained below 0.004 nmol/mg-DCW, regardless of malonate supplementation (Figure 2a). In contrast, malonate supplementation increased the intracellular malonyl-CoA concentration in the BLmat strain to 0.135 nmol/mg-DCW, corresponding to a 33-fold increase (Figure 2a). Supplemented malonate remained almost unchanged in the culture supernatant of BL21(DE3) over 72 h, whereas its concentration decreased to 0.3 mM in the BLmat strain, indicating that approximately 99% of the supplemented malonate was consumed (Figure 2b). No extracellular malonyl-CoA was detected in the culture supernatant throughout the experiment. These results indicate that the MatB–MatC module effectively enhanced intracellular malonyl-CoA availability in *E. coli* when malonate was supplied as an external substrate.

### 3.2. Heterologous Expression of MatB and MatC Enhanced RK Production in RK-Producing Recombinant E. coli in the Presence of Malonate

**Figure 3:**
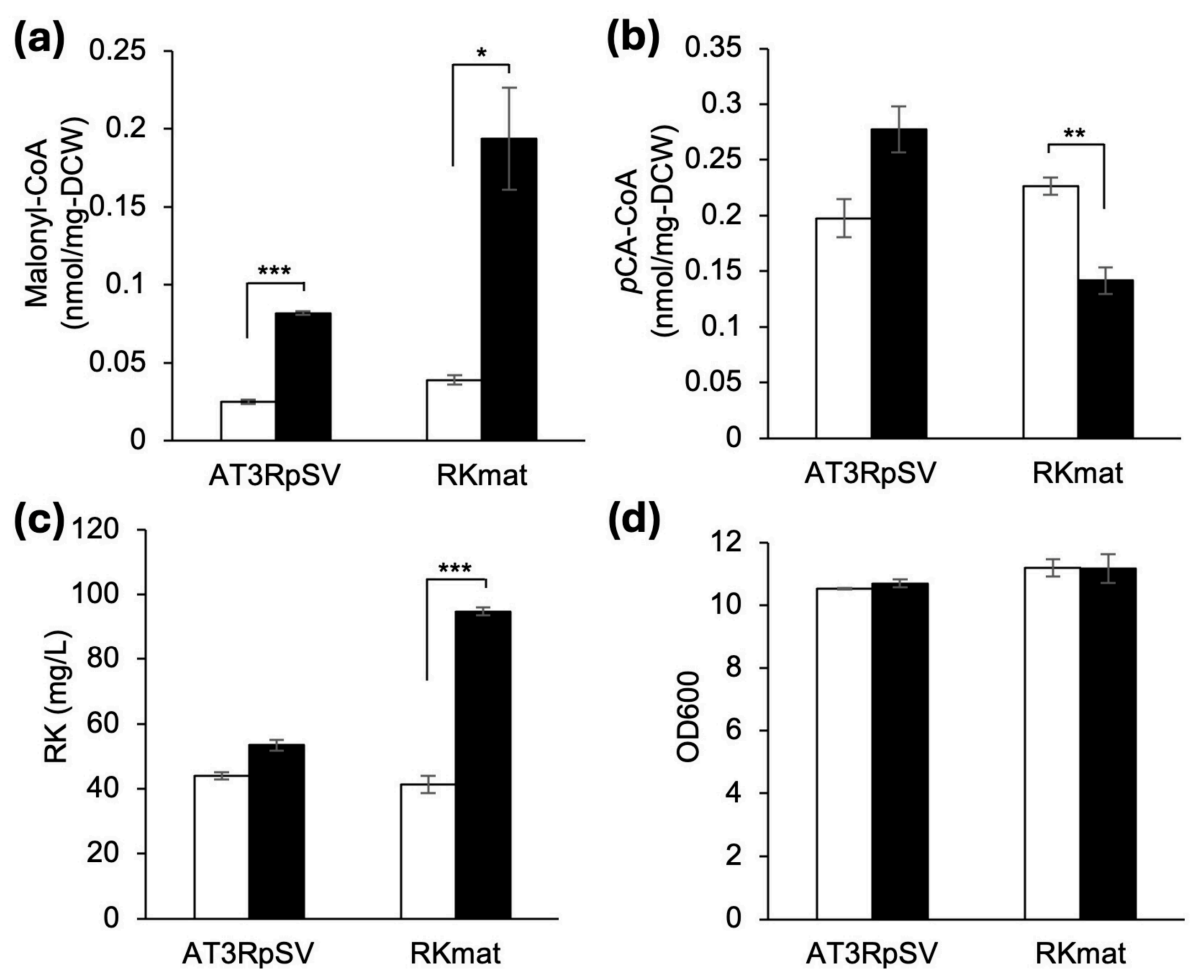
Effects of the MatB–MatC module and malonate supplementation on intracellular acyl-CoA levels and RK production in the RK-producing strain. The RK-producing *E. coli* strain AT3RpSV and the *matBC*-expressing derivative RKmat were cultured in fermentation medium with or without 50-mM malonate. (**a**) Intracellular malonyl-CoA levels. (**b**) Intracellular *p*CA-CoA levels. (**c**) RK production. (**d**) Final OD600 values. White and black bars indicate cultivation without and with malonate supplementation, respectively. Values represent the mean ± standard deviation of three independent biological replicates. Statistical significance was evaluated using Welch’s *t*-test for the indicated comparisons. One, two, and three asterisks indicate *p* < 0.05, *p* < 0.01, and *p* < 0.001, respectively. AT3RpSV, *E. coli* strain harboring the artificial RK biosynthetic pathway; DCW, dry cell weight; *p*CA-CoA, *p*-coumaroyl-CoA; RK, raspberry ketone; RKmat, *matB*/*matC*-expressing RK-producing *E. coli* strain.

We introduced the *matBC* co-expression plasmid into AT3RpSV, an *E. coli* strain harboring the artificial RK biosynthetic pathway [12], to generate RKmat. AT3RpSV and RKmat were cultured in shake flasks using fermentation medium with or without 50-mM malonate. Malonate supplementation increased intracellular malonyl-CoA levels in both AT3RpSV and RKmat, with the highest accumulation observed in RKmat supplemented with 50-mM malonate (Figure 3a). The intracellular level of *p*CA-CoA, another BAS substrate, decreased in RKmat upon malonate supplementation (Figure 3b). This decrease was accompanied by an increase in RK production to 94.7 mg/L, corresponding to a 2.3-fold increase compared with the condition without malonate (Figure 3c). These results indicate that the MatB–MatC module functioned in the RK-producing strain and that its introduction enhanced RK production in the presence of malonate. To evaluate whether MatB–MatC expression and malonate supplementation affected cell growth under RK-producing conditions, we compared the final OD600 values of AT3RpSV and RKmat cultures with or without 50-mM malonate supplementation. No significant decrease in final OD600 was observed in RKmat supplemented with malonate compared with the corresponding control conditions (Figure 3d). These results suggest that MatB–MatC-dependent malonyl-CoA supply did not cause severe growth inhibition under the tested flask cultivation conditions.

### 3.3. Metabolic Profiling-Guided Optimization of Malonate Supplementation for Enhanced RK Production

**Figure 4:**
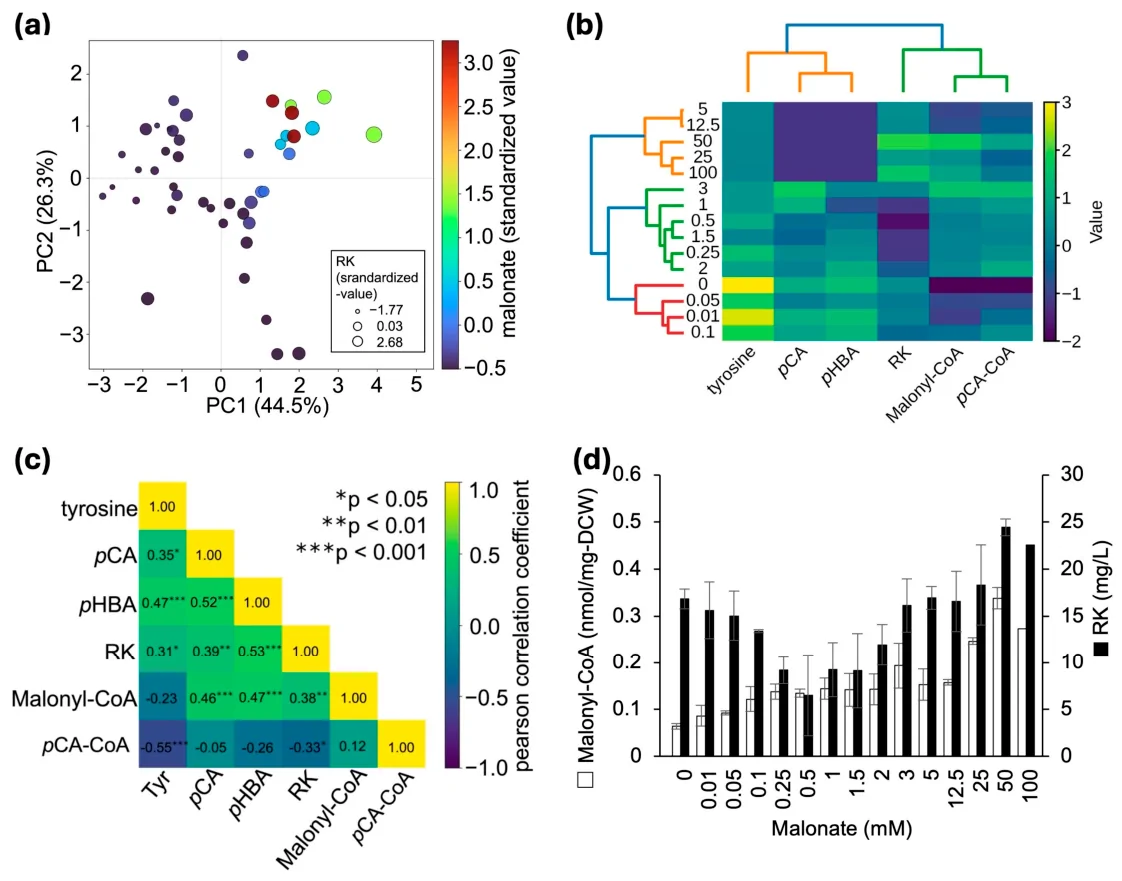
Effects of malonate supplementation on metabolite profiles, intracellular malonyl-CoA levels, and RK production. The RKmat strain was cultured in 96-deep-well plates using fermentation medium supplemented with different concentrations of malonate. Extracellular tyrosine, pCA, *p*-hydroxybenzalacetone, and RK, as well as intracellular *p*CA-CoA and malonyl-CoA, were quantified by LC–MS. (**a**) PCA of metabolite profiles. The color and size of each symbol indicate the standardized malonate value and RK production level, respectively. (**b**) Hierarchical clustering heatmap of standardized metabolite levels at each malonate concentration. (**c**) Pearson’s correlation matrix of metabolite levels and RK production. One, two, and three asterisks indicate *p* < 0.05, *p* < 0.01, and *p* < 0.001, respectively. (**d**) Intracellular malonyl-CoA levels and RK production at each malonate concentration. White and Black Bars indicate intracellular malonyl-CoA levels and RK production, respectively. Values represent the mean ± standard deviation of three independent biological replicates. DCW, dry cell weight; *p*CA, *p-*coumaric acid; PCA, principal component analysis; *p*CA-CoA, *p*-coumaroyl-CoA; *p*HBA, *p*-hydroxybenzalacetone; RK, raspberry ketone; RKmat, *matB*/*matC*-expressing RK-producing *E. coli* strain.

To optimize malonate supplementation for RK production and investigate its effects on RK pathway metabolites, the RKmat strain was cultured in 96-deep-well plates using fermentation medium supplemented with malonate at final concentrations ranging from 0.01 to 100 mM. Extracellularly accumulated tyrosine, *p*CA, *p*HBA, and RK, as well as intracellular *p*CA-CoA and malonyl-CoA, were quantified by LC–MS. The resulting metabolite profiles were analyzed by PCA, hierarchical clustering, and correlation analysis (Figure 4a–c). The processed data matrices used for these analyses are provided in Tables S1 and S2. PCA revealed that the metabolite profiles were separated along PC1 and PC2, which explained 44.5% and 26.3% of the total variance, respectively (Figure 4a). Samples with higher malonate levels were distributed toward the positive side of PC1, where RK production also tended to be higher. Hierarchical clustering of the metabolite profiles separated the samples largely according to the malonate supplementation level (Figure 4b). Low or no malonate supplementation clustered together and was characterized by relatively high tyrosine accumulation and low RK and malonyl-CoA levels. Higher malonate concentrations formed a distinct cluster associated with increased RK and malonyl-CoA levels. Correlation analysis showed that RK production was positively correlated with *p*HBA, *p*CA, and malonyl-CoA, whereas it was negatively correlated with intracellular *p*CA-CoA (Figure 4c). Among these metabolites, *p*HBA showed the strongest positive correlation with RK.

Among the tested malonate concentrations, supplementation with 50-mM malonate resulted in the highest RK production, reaching 24.43 mg/L, with intracellular malonyl-CoA accumulating to 0.338 nmol/mg-DCW (Figure 4d). Therefore, 50-mM malonate was selected as the optimal supplementation concentration for subsequent experiments.

### 3.4. Optimization and Scale-Up of Fed-Batch Cultivation for Enhanced RK Production

**Figure 5:**
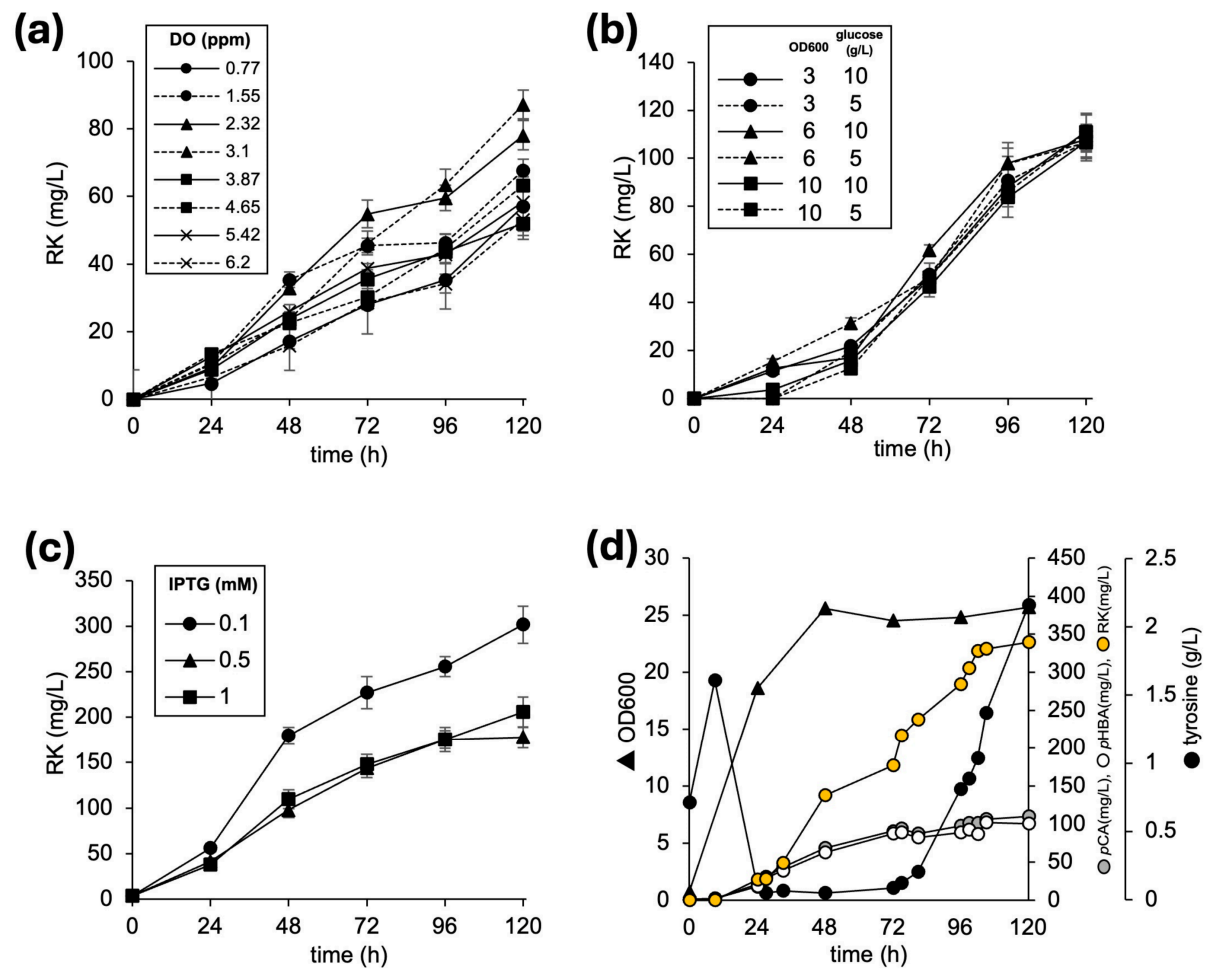
Optimization and scale-up of fed-batch cultivation for RK production by the RKmat strain. Cultivations were performed in jar fermenters using fermentation medium supplemented with 50-mM malonate at 28 °C and pH 7.0. The pH was maintained by adding 10% ammonia solution. (**a**–**c**) Effects of DO concentration (**a**), OD_600_ at IPTG induction and feedback-controlled glucose concentration (**b**), and IPTG concentration (**c**) on RK production. Values in panels (**a**–**c**) represent the mean ± standard deviation of three independent biological replicates. (**d**) Time-course profile of cell growth, glucose concentration, pathway intermediates, and RK production during a 2-L jar fermenter cultivation with a 1-L working volume under the optimized conditions. The time-course data shown in panel (**d**) were obtained from a single scale-up cultivation. IPTG was added to a final concentration of 0.1 mM at an OD_600_ of 3, and the glucose concentration was maintained at approximately 10 g/L. DO was maintained at 3.1 ppm throughout the cultivation. DO, dissolved oxygen; IPTG, isopropyl β-D-thiogalactoside; OD_600_, optical density at 600 nm; RK, raspberry ketone; RKmat, *matB*/*matC*-expressing RK-producing *E. coli* strain.

Using a 100-mL jar fermenter, we optimized fed-batch cultivation conditions for RK production by varying the DO concentration, induction timing, glucose concentration, and IPTG concentration. The molar yields reported in this section are glucose-based molar yields calculated from glucose consumption and therefore do not include the carbon contribution from externally supplied malonate. RK production was highest at a DO concentration of 3.1 ppm, reaching 87.9 mg/L with a glucose-based molar yield of 0.86% (Figure 5a). Under this DO condition, RK production was comparable among the tested induction timing and glucose-feeding conditions (Figure 5b). The condition in which IPTG was added at an OD600 of 3 and the glucose concentration was maintained at approximately 10 g/L produced 111.2 mg/L RK with a glucose-based molar yield of 1.31% and was selected for subsequent IPTG optimization. Further optimization of the IPTG concentration showed that induction using 0.1 mM IPTG increased RK production to 301.4 mg/L, corresponding to a glucose-based molar yield of 2.10% (Figure 5c). The optimized conditions were therefore defined as induction using 0.1-mM IPTG at an OD_600_ of 3, a DO concentration of 3.1 ppm, and a glucose concentration of approximately 10 g/L.

Scale-up cultivation under the optimized conditions in a 2-L jar fermenter with a 1-L working volume produced 339.6 mg/L RK after 120 h, corresponding to a glucose-based molar yield of 3.41% and an overall volumetric productivity of 2.83 mg L^−1^ h^−1^ (Figure 5d). The time-course profile obtained from this single 2-L cultivation showed that RK accumulation was relatively slow during the early growth phase, increased more rapidly after substantial biomass accumulation, and then gradually plateaued during the later phase. Based on this profile, the apparent volumetric productivity was 13.2 mg L^−1^ h^−1^ during 72–75 h and decreased to 0.619 mg L^−1^ h^−1^ during 105–120 h. A similar RK accumulation pattern was also observed in independent 100-mL jar fermenter cultivations under the selected production condition (Figure S1), suggesting that the phase-dependent trend observed in the 2-L cultivation was consistent with the trend observed at the 100-mL scale.

## 4. Discussion

In this study, we demonstrated that the MatB–MatC-mediated malonate assimilation pathway enhanced intracellular malonyl-CoA availability and improved de novo RK production in engineered *E. coli*. Heterologous expression of *matB* and *matC* enabled externally supplied malonate to be converted into intracellular malonyl-CoA, whereas malonate supplementation alone had little effect in BL21(DE3) without *matB* and *matC* expression. These results confirm that the MatB–MatC module functioned as a malonate-dependent malonyl-CoA supply system in *E. coli* (Figure 2).

Increasing malonyl-CoA availability has been widely recognized as an important strategy for improving microbial production of polyketide-derived compounds because malonyl-CoA is a central precursor for fatty acids, flavonoids, stilbenoids, and various polyketides [14]. In RK biosynthesis, BAS catalyzes the condensation of *p*CA-CoA with malonyl-CoA to form *p*HBA; therefore, the availability of these two acyl-CoA substrates is expected to strongly influence RK pathway flux [19,31]. Previous studies on RK-producing *E. coli* have shown that enhancement of *p*CA-CoA formation increases RK production [12]. Cerulenin supplementation can further improve RK production by increasing intracellular malonyl-CoA levels [12]. However, cerulenin inhibits fatty acid biosynthesis and is less suitable for scalable fermentation because of its high cost and inhibitory effect [27,32,33,34]. In this context, the *matBC*-based strategy provides a direct and tunable alternative for increasing malonyl-CoA availability through malonate supplementation.

To clarify the novelty and performance of the present system, representative microbial RK production studies are summarized in Table 4. Because previous studies differ in host strain, substrate or precursor, cultivation mode, and yield definition, direct comparison of titers, yields, and productivities should be interpreted with caution. Nevertheless, the present study is distinguished from previous studies by combining de novo RK biosynthesis, MatB–MatC-dependent malonyl-CoA supply, pathway-level metabolite profiling, and controlled fed-batch optimization.

Previous studies have shown that RK production can be improved through pathway engineering, optimization of enzyme expression, enhancement of precursor availability, and MatB–MatC-dependent malonyl-CoA supply [15,35,36]. Building on these studies, we quantitatively evaluated and optimized a malonate-dependent malonyl-CoA supply module in a de novo RK-producing strain under cerulenin-free conditions. Multiple pathway-related metabolites were systematically analyzed together with RK production under various malonate supplementation conditions to examine how MatB–MatC-dependent malonyl-CoA supply was associated with metabolite distribution within the RK biosynthetic pathway. Furthermore, the optimized production strategy was evaluated under controlled fed-batch cultivation conditions at a larger scale. Thus, the present study complements previous RK production studies by examining both pathway-level metabolic changes and fermentation performance in an engineered *E. coli* system.

Introduction of the MatB–MatC module into the RK-producing strain enhanced RK production in the presence of malonate (Figure 3a,c). Malonate supplementation increased intracellular malonyl-CoA levels in the RK-producing background, with the highest accumulation observed in RKmat supplemented with malonate. Nevertheless, intracellular *p*CA-CoA levels did not increase in RKmat upon malonate supplementation, indicating that the increased RK production was associated with enhanced malonyl-CoA availability rather than increased *p*CA-CoA accumulation. Correlation analysis further showed that RK production was more strongly correlated with *p*HBA than with malonyl-CoA (Figure 4c), suggesting that downstream pathway steps, including *p*HBA formation and conversion to RK, also influenced final RK production.

The final OD600 values obtained under RK-producing conditions showed that MatB–MatC expression and 50-mM malonate supplementation did not substantially reduce cell growth (Figure 3d). This suggests that the enhanced RK production was not simply due to differences in biomass accumulation and that severe growth inhibition was not observed under the tested conditions. However, final OD600 alone is not sufficient to fully evaluate metabolic burden. Because malonyl-CoA is closely linked to fatty acid biosynthesis and central carbon metabolism, elevated malonyl-CoA may still affect carbon partitioning, substrate consumption, by-product formation, or growth kinetics. Future studies should therefore include growth curve analysis, substrate consumption profiles, by-product profiling, and carbon balance analysis.

Metabolic profiling further highlighted the importance of optimizing malonate supplementation. PCA and hierarchical clustering showed that malonate supplementation broadly shifted the RK pathway metabolite profile, separating low-malonate and high-malonate conditions (Figure 4a,b). Among the tested concentrations, 50-mM malonate resulted in the highest RK production (Figure 4d). This finding indicates that malonate supplementation should be optimized rather than simply maximized, because increased precursor supply does not necessarily lead to proportional increases in final product formation. Although most of the supplemented malonate was consumed in the MatB–MatC validation experiment, RK production in the RK-producing strain did not increase proportionally with increasing malonate supplementation. This suggests that malonate-derived carbon may not have been directed exclusively toward RK biosynthesis. The accumulation of malonyl-CoA under some malonate-supplemented conditions further indicates that the additional malonyl-CoA generated by MatB–MatC was not fully converted into RK. This may be explained by limited availability of the second BAS substrate, *p*CA-CoA, limited BAS catalytic capacity, insufficient conversion of *p*HBA to RK, or competition with native cellular processes such as fatty acid biosynthesis. Thus, pathway-level metabolic profiling provides useful information for optimizing precursor-supply modules in engineered biosynthetic systems, where precursor availability, pathway enzyme activity, cofactor supply, and product formation must be balanced.

Optimization of fed-batch cultivation further improved RK production. By controlling the DO concentration, induction timing, glucose concentration, and IPTG concentration, RK production in the 100-mL jar fermenter reached 301.4 mg/L (Figure 5a–c). Under the optimized conditions, scale-up cultivation in a 2-L jar fermenter with a 1-L working volume produced 339.6 mg/L RK (Figure 5d). In the 2-L cultivation, tyrosine accumulated markedly during the later phase, when RK accumulation gradually plateaued. A similar trend was observed in independent 100-mL jar fermenter cultivations under the selected production condition (Figure S1), supporting an association between late-stage tyrosine accumulation and the reduced RK accumulation rate. These results suggest that aromatic precursor supply itself was unlikely to be the primary limitation during the later phase; instead, conversion of tyrosine to downstream phenylpropanoid intermediates, *p*CA-CoA formation, BAS-catalyzed condensation, or subsequent conversion of *p*HBA to RK may have limited late-stage RK production.

The glucose-based molar yield also increased from 2.10% in the optimized 100-mL jar fermenter to 3.41% in the 2-L jar fermenter. This increase may reflect improved process control in the larger fermenter, including more stable aeration, mixing, pH control, glucose feeding, and DO regulation, which could have improved the balance between cell growth, pathway expression, precursor supply, and RK formation. However, the present data do not allow us to determine which process parameter was primarily responsible for the increased yield. Therefore, this improvement should be interpreted as an observed outcome under the tested scale-up conditions, and further controlled process analysis will be required to identify the underlying factors.

In addition, the yields reported in this study were calculated as glucose-based molar yields and did not include the carbon contribution from externally supplied malonate. Therefore, these values should not be interpreted as overall substrate-to-product carbon conversion efficiencies. Because malonate uptake was not monitored throughout all RK production and fed-batch conditions, overall carbon yield including malonate was not calculated in this study. Despite these limitations, the 2-L jar fermenter result supports the feasibility of applying the optimized malonate-dependent RK production system under larger-scale controlled cultivation conditions. Future optimization should include direct analysis of malonate consumption, by-product formation, and carbon balance, as well as balancing the expression of RK pathway enzymes, improving malonate uptake and utilization efficiency, enhancing conversion of *p*HBA to RK, and reducing competing metabolic fluxes.

Together, these findings support MatB–MatC-dependent malonyl-CoA supply as an effective strategy for improving de novo RK production in *E. coli*. This study also highlights the value of pathway-level metabolic profiling and fermentation optimization for improving microbial production of malonyl-CoA-derived natural products.

## Figures and Tables

**Table 1:** Strains used in this study.

*E. coli* Strains	Relevant Genotype	Reference
BL21(DE3)	F^−^ *ompT hsdS*_B_(r_B_^−^m_B_^−^) *gal dcm* λ(DE3)	Novagen
BLmat	BL21(DE3) harboring pET-matB-matC	This study
TY	BL21(DE3) *ΔtyrR*::Zeo^r^	[12]
AT3RpSV	TY harboring pET-tyrA/pACYC-aroG4/pRSF-Rgpal/pCDF-AtuCL-RpBAS^S338gV^-fabF	[12]
RKmat	TY harboring pET-matB-matC/pACYC-aroG4/pRSF-Rgpal-tyrA/pCDF-AtuCL-RpBAS^S338V^-fabF	This study

**Table 2:** Plasmids used in this study.

Plasmid	Description	Reference
pET-matB-matC	*matB* and *matC* under the control of T7 promoter (Amp^r^)	This study
pRSF-Rgpal-tyrA	*Rgpal* (PAL gene from *Camellia sinensis*) and *tyrA* under the control of T7 promoter (Km^r^)	This study
pET-28a-AtuCL_Nterm	*AtuCL* (CL gene from *Agrobacterium tumefaciens* C58) with an N-terminal His_6_ tag	This study

**Table 3:** List of primers.

Name	Nucleotide Sequence (5′-3′)	Used to Generate
matB_fw	ACTTTAAGAAGGAGATATACCATGGTATCAAATCACCTA	*matB* (pET-matB construction)
matB_rv	TTAAGCATTATGCGGCCGCATTAGGTACGGGTGTACAGG T	
matC_fw	GTATAAGAAGGAGATATACATATGGGGATTGAATTGCTA	*matC* (pET-matB-matC construction)
matC_rv	CAGCGGTTTCTTTACCAGACTTACTTACACCAGACCCGG CAC	
tyrA_fw	GCTGCTGCCAATGGTTGCTGAATTGACC	Partial region of pET-tyrA (pRSF-Rgpal-tyrA construction)
tyrA_rv	CTAGGTTAATTTACTGGCGGTTGTCATTC	
AtuCL_fw2_Nterm	CTGGTGCCGCGCGGCAGCCATATGAGTGTGAAACTTTGGTC	Partial region of pRSF-AtuCL (pET-28a-CoA-ligase construction)
AtuCL_rv2_Nterm	AGTGGTGGTGGTGGTGGTGCTCGAGTCATGCTGCTACCTCTCTG	

**Table 4:** Comparison of representative microbial raspberry ketone production systems.

Host	Production Mode	Engineering/Strategy	Main Substrate or Precursor	Cultivation Mode	RK Titer	Yield	Productivity	Key Feature	Reference
*S. cerevisiae* (wine yeast)	De novo and precursor-assisted biosynthesis	PAL/TAL, C4H, 4CL and BAS; synthetic 4CL–BAS fusion	Grape juice sugars; or *p*-coumaric acid	Flask/wine fermentation	3.5 mg/L (de novo); 7.5 mg/L (+ *p*-coumaric acid)	NR	NR	First de novo RK production in an industrial yeast; enzyme fusion improved pathway flux	[4]
*E. coli* BL21(DE3)	Precursor-based biosynthesis	Balanced expression of 4CL, BAS and RZS1	*p*-Coumaric acid	Flask/fermentation	90.97 mg/L	NR	NR	Near-complete reduction of *p*-hydroxybenzalacetone and optimized pathway-module expression	[15]
*C. glutamicum*	Precursor-based biosynthesis	4CL, BAS and CurA with newly identified benzalacetone reductase activity	*p*-Coumaric acid	Flask/fermentation	99.8 mg/L	NR	NR	Alternative bacterial host with comparatively high tolerance to phenylbutanoids	[14]
*E. coli* CR8	Fatty-acid-assisted/precursor-fed biosynthesis	Fatty-acid utilization chassis, fatty-acid-responsive promoter engineering and pntAB expression	Soybean oil + glycerol + fed *p*-coumaric acid	1-L fed-batch bioconversion	180.94 mg/L	NR	NR	Alternative feedstock route supporting acetyl-CoA/malonyl-CoA and reducing-power supply	[35]
*E. coli* DH10β	De novo/precursor-assisted synthetic pathway	EcoFlex pathway refactoring; promoter/RBS balancing; MatB-mediated malonyl-CoA regeneration	L-Tyrosine + malonate in rich medium	Flask/fermentation	12.9 mg/L	NR	NR	Design–build–test–learn optimization and explicit use of MatB for malonyl-CoA regeneration	[13]
*E. coli* BL21(DE3)	De novo biosynthesis	Aromatic precursor engineering, FabF overexpression and cerulenin-mediated malonyl-CoA enhancement	Glucose	1-L jar fed-batch fermentation	62 mg/L	0.12% vs. glucose	NR	First controlled fed-batch de novo RK production from glucose in *E. coli*	[12]
*S. cerevisiae*	De novo biosynthesis	Modular metabolic engineering and synthetic coculture strategies	Glucose	50-mL flask fermentation	63.5 mg/L	2.1 mg/g glucose	NR	Highest reported yeast de novo titer at publication; modular division of pathway functions	[36]
*E. coli* RKmat (BL21(DE3)-derived)	De novo biosynthesis with malonate-dependent malonyl-CoA supply	matB/matC expression, malonate optimization, pathway-metabolite profiling and fed-batch optimization	Glucose + malonate	2-L jar fermenter	339.6 mg/L	3.41% glucose-based molar yield *	2.83 mg L^−1^ h^−1^ overall; 13.2 mg L^−1^ h^−1^ max. apparent phase productivity *	Scale-up under controlled fed-batch conditions with improved apparent glucose-based molar yield	This study

NR, not reported. Values are shown as reported in the original studies. Direct comparison should be made cautiously because host strains, substrates/precursors, cultivation modes, process durations, and yield definitions differ. * The apparent glucose-based molar yields in this study do not include the carbon contribution from externally supplied malonate. Overall volumetric productivity was calculated as final RK titer divided by total cultivation time. Maximum apparent phase productivity was calculated from the RK concentration difference between adjacent sampling points.

## Data Availability

The original contributions presented in this study are included in the article and Supplementary Material. Further inquiries can be directed to the corresponding author.

## Supplementary Materials

The following supporting information can be downloaded at https://www.mdpi.com/article/10.3390/biotech15030071/s1, Figure S1: Time-course profiles of cell growth, Tyr accumulation, and RK production by the RKmat strain during optimized fed-batch cultivation in 100-mL jar fermenters. IPTG was added to a final concentration of 0.1 mM at an OD600 of 3, and the glucose concentration was maintained at approximately 10 g/L. DO was maintained at 3.1 ppm throughout the cultivation. Black triangles, yellow circles, and black circles indicate OD600 values, RK concentrations, and Tyr concentrations, respectively. Values represent the mean ± standard deviation of three independent biological replicates. The apparent volumetric productivity of RK was calculated as the change in the mean RK concentration divided by the corresponding cultivation interval. The apparent volumetric productivities during 72–75 h and 105–120 h were approximately 9.18 and 1.86 mg L^−1^ h^−1^, respectively. DO, dissolved oxygen; IPTG, isopropyl β-D-thiogalactoside; OD600, optical density at 600 nm; RK, raspberry ketone; RKmat, matB/matC-expressing RK-producing *E. coli* strain; Table S1: List of processed data matrix used for principal component analysis and correlation analysis; Table S2: List of processed data matrix used for hierarchical clustering.

## Abbreviations

The following abbreviations are used in this manuscript.

ACC	acetyl-CoA carboxylase
ATP	adenosine triphosphate
AtuCL	*p*-coumaric acid CoA ligase
BAR	benzalacetone reductase
BAS	benzalacetone synthase
CoA	coenzyme A
DCW	dry cell weight
DO	dissolved oxygen
HPLC	high-performance liquid chromatography
IPTG	isopropyl β-D-thiogalactoside
LB	Lysogeny Broth
LC	liquid chromatography
LC–ESI–MS/MS	liquid chromatography–electrospray ionization–tandem mass spectrometry
LC–MS	liquid chromatography–mass spectrometry
MOPS	3-(N-morpholino)propanesulfonic acid
MS	mass spectrometry
OD_600_	optical density at 600 nm
*p*CA	*p*-coumaric acid
*p*CA-CoA	*p*-coumaroyl-CoA
PCR	polymerase chain reaction
PCA	principal component analysis
*p*HBA	*p*-hydroxybenzalacetone
RgPAL	phenylalanine/tyrosine ammonia-lyase
RK	raspberry ketone
RpBAS^S338V^	benzalacetone synthase variant S338V
