# Supplementary material for: Optimizing MatB–MatC-Dependent Malonyl-CoA Supply for Enhanced Raspberry Ketone Production in Escherichia coli
Source: BioTech (Basel). 2026 Aug 21;15(3):71. doi: 10.3390/biotech15030071 (PMC13511146; doi:10.3390/biotech15030071)
Supplement: Supplementary file 1 [file biotech-15-00071-s001.zip › biotech-4425765 - Figure S1.pdf]

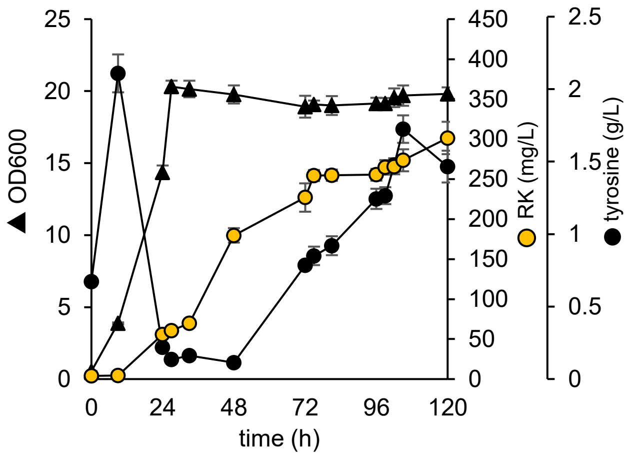

Figure S1. Time-course profiles of cell growth, Tyr accumulation, and RK production by the RKmat strain during optimized fed-batch cultivation in 100-mL jar fermenters. IPTG was added to a final concentration of 0.1 mM at an OD600 of 3, and the glucose concentration was maintained at approximately 10 g/L. DO was maintained at 3.1 ppm throughout the cultivation. Black triangles, yellow circles, and black circles indicate OD600 values, RK concentrations, and Tyr concentrations, respectively. Values represent the mean ± standard deviation of three independent biological replicates.

The apparent volumetric productivity of RK was calculated as the change in the mean RK concentration divided by the corresponding cultivation interval. The apparent volumetric productivities during 72–75 h and 105–120 h were approximately 9.18 and 1.86 mg L^{-1} h^{-1}, respectively.

DO, dissolved oxygen; IPTG, isopropyl β-D-thiogalactoside; OD600, optical density at 600 nm; RK, raspberry ketone; RKmat, matB/matC-expressing RK-producing *E. coli* strain.
